# Development and Application of an Open Tool for Sharing and Analyzing Integrated Clinical and Environmental Exposures Data: Asthma Use Case

**DOI:** 10.2196/32357

**Published:** 2022-04-01

**Authors:** Karamarie Fecho, Stanley C Ahalt, Stephen Appold, Saravanan Arunachalam, Emily Pfaff, Lisa Stillwell, Alejandro Valencia, Hao Xu, David B Peden

**Affiliations:** 1 Renaissance Computing Institute University of North Carolina at Chapel Hill Chapel Hill, NC United States; 2 Kenan-Flagler Business School University of North Carolina at Chapel Hill Chapel Hill, NC United States; 3 Institute for the Environment University of North Carolina at Chapel Hill Chapel Hill, NC United States; 4 North Carolina Translational and Clinical Sciences Institute University of North Carolina at Chapel Hill Chapel Hill, NC United States; 5 Division of Allergy & Immunology, Department of Pediatrics School of Medicine University of North Carolina at Chapel Hill Chapel Hill, NC United States; 6 Center for Environmental Medicine, Asthma and Lung Biology School of Medicine University of North Carolina at Chapel Hill Chapel Hill, NC United States

**Keywords:** open patient data, electronic health records, airborne pollutant exposures, socioeconomic exposures, medication exposures, asthma exacerbation

## Abstract

**Background:**

The Integrated Clinical and Environmental Exposures Service (ICEES) serves as an open-source, disease-agnostic, regulatory-compliant framework and approach for openly exposing and exploring clinical data that have been integrated at the patient level with a variety of environmental exposures data. ICEES is equipped with tools to support basic statistical exploration of the integrated data in a completely open manner.

**Objective:**

This study aims to further develop and apply ICEES as a novel tool for openly exposing and exploring integrated clinical and environmental data. We focus on an asthma use case.

**Methods:**

We queried the ICEES open application programming interface (OpenAPI) using a functionality that supports chi-square tests between feature variables and a primary outcome measure, with a Bonferroni correction for multiple comparisons (α=.001). We focused on 2 primary outcomes that are indicative of asthma exacerbations: annual emergency department (ED) or inpatient visits for respiratory issues; and annual prescriptions for prednisone.

**Results:**

Of the 157,410 patients within the asthma cohort, 26,332 (16.73%) had 1 or more annual ED or inpatient visits for respiratory issues, and 17,056 (10.84%) had 1 or more annual prescriptions for prednisone. We found that close proximity to a major roadway or highway, exposure to high levels of particulate matter ≤2.5 μm (PM_2.5_) or ozone, female sex, Caucasian race, low residential density, lack of health insurance, and low household income were significantly associated with asthma exacerbations (*P*<.001). Asthma exacerbations did not vary by rural versus urban residence. Moreover, the results were largely consistent across outcome measures.

**Conclusions:**

Our results demonstrate that the open-source ICEES can be used to replicate and extend published findings on factors that influence asthma exacerbations. As a disease-agnostic, open-source approach for integrating, exposing, and exploring patient-level clinical and environmental exposures data, we believe that ICEES will have broad adoption by other institutions and application in environmental health and other biomedical fields.

## Introduction

Several large-scale initiatives are advancing efforts to reduce barriers surrounding access to patient data maintained in electronic health record (EHR) systems. Relevant initiatives include Columbia Open Health Data [1] and Medical Information Mart for Intensive Care [2]. The common goal is to promote open access to and sharing of patient data for research purposes, while respecting and preserving patient privacy and institutional assurances.

As part of the Biomedical Data Translator program (Translator) [3,4], supported by the National Center for Advancing Translational Sciences, we have developed a disease-agnostic, regulatory-compliant framework and approach for openly exposing and exploring patient data: the Integrated Clinical and Environmental Exposures Service (ICEES) [5]. ICEES was designed to overcome the regulatory, cultural, and technical challenges that hinder efforts to openly share and explore patient data [6,7]. ICEES is unique from similar efforts toward open patient data in that the service provides access to clinical data that have been integrated at the patient level with environmental exposures data derived from a variety of public sources. Thus, ICEES allows for patient-level research in environmental health and related fields.

Herein, we describe the further development and application of ICEES to data on a large cohort of patients with a diagnosis of asthma or a related condition. We examine the impact of select airborne pollutant exposures, demographic factors, and socioeconomic exposures on asthma exacerbations, which we define using 2 primary outcome measures: annual emergency department (ED) or inpatient visits for respiratory issues and annual prescriptions for prednisone. We present our findings and compare results for the 2 outcome measures.

## Methods

### Study Approval

All study procedures were approved by the Institutional Review Board at the University of North Carolina at Chapel Hill (protocol #16-2978). Informed consent was not required as the study involved existing biomedical data only and patient contact was not involved.

### Asthma Cohort

ICEES was designed as a disease-agnostic, regulatory-compliant, open platform. For the work described here, we focused on 157,410 patients with asthma or a related pulmonary condition at UNC Health (all available sites). The specific criteria used to select patients for inclusion in the ICEES asthma cohort were adapted from [8] and included a combination of diagnoses, medications, and laboratory measures. (Details can be found in [5].) Briefly, we captured data on (1) patients with a diagnostic code for “asthma” and prescribed or administered medications that are typically used to treat asthma; (2) patients with a diagnostic code for a respiratory condition other than asthma and prescribed or administered medications that are typically used to treat asthma; (3) patients with a diagnostic code for a pulmonary condition other than asthma but prescribed tests or procedures that are typically used to manage asthma; and (4) patients with a diagnostic code for a respiratory condition other than asthma but with frequent ED visits in which albuterol nebulizer treatments were administered.

### ICEES Integrated Feature Tables

“ICEES integrated feature tables” are key to the open design of ICEES. These tables were created using a complex custom software pipeline within a secure environment and under a protocol (#16-2978) approved by the Institutional Review Board at the University of North Carolina at Chapel Hill. For data extraction, Clinical Asset Mapping Program for Health Level 7 Fast Healthcare Interoperability Resource (CAMP FHIR) converted patient data from the PCORnet common data model to FHIR files [9]. FHIR Patient data Integration Tool (FHIR PIT) then ingested the FHIR files and integrated the patient data with multiple sources of environmental exposures data, using patient geocodes as reported in the EHR and dates [10]. The exposures data were derived from public sources and included airborne pollutant exposures data from the United States (US) Environmental Protection Agency Fused Air Quality Surface Using Downscaling repository; major roadway or highway exposures data (a proxy for airborne pollutant exposures) from the US Department of Transportation; and socioeconomic exposures data from the US Census Bureau American Community Survey. (Additional information on the sources of environmental exposures data can be found in [11].) After the data were integrated, the resultant ICEES integrated feature tables were stripped of identifiers per the Safe Harbor method outlined in the Health Insurance Portability and Accountability Act (HIPAA) before being exposed with an open application programming interface (OpenAPI).

ICEES integrated feature tables were created with respect to 1-year “study” periods, that is, calendar years, to provide a reference point for date-based calculations such as age and estimated exposure. Rows contained binned or recoded data on individual patients, with column headers representing data fields for each of the integrated feature variables. Of note, our institution classifies exposure estimates as “secondary protected health information” because the estimates are derived using primary protected health information (PHI; namely, geocodes and dates) to account for the fact that exposure estimates vary across space and time. We addressed this concern by binning all exposure estimates.

The binning strategy that was applied to each feature variable was based on a combination of expert opinion, published literature, and mathematical approaches. Age on day 1 of the 1-year study period was binned using our prior approach [5,12,13]: <5, 5-17, 18-44, 45-64, and 65-89 years (89 years being the oldest permissible age per HIPAA). Sex was treated as male or female as coded in the EHR. Multiple race categories were available in ICEES; we focused on Caucasian and African American, as each of the other categories encompassed ≤1% of the total patients. Rural versus urban residence was examined using the US Census Bureau classifications based on American Community Survey–estimated residential density: rural area (<2500 persons per Census block group); urban cluster (between 2500 and 50,000 persons per Census block group); and urbanized area (>50,000 persons per Census block group). Estimated probability of no health insurance and estimated median household income were binned using the pandas.qcut function, which bins according to frequencies: [0, 0.637], [0.0637, 0.1121], [0.1121, 0.1644], [0.1644, 0.5548] estimated probability of no health insurance; [7,470, 36,635], [36,635, 46,750], [46,750, 59,566], [59,566, 78,355], [78,355, 250,001] estimated median household income (US $). Proximity to a major roadway or highway was binned based on published work [14]: 0-49, 50-99, 100-149, 150-199, 200-249, ≥250 m). Average daily particulate matter ≤2.5 μm (PM_2.5_) and maximum daily ozone exposure were averaged over the 1-year study period and binned using the pandas.cut function, which bins patients according to the distribution of value estimates: [3.27, 6.30], (6.30, 7.81], (7.81, 10.83] μg/m^3^ PM_2.5_; [27.80, 39.00], (39.00, 42.73], (42.73, 46.45] ppb ozone. Importantly, bins were determined in our prior work to be sufficiently granular for statistical analysis [5].

### ICEES OpenAPI

We accessed the ICEES OpenAPI through the ICEES Swagger OpenAPI interface and by command-line requests. An ICEES user interface was also available. ICEES was designed to support several functionalities for exploring and displaying the data, including chi-square tests, with counts of patients, chi-square statistics, and probabilities returned to users. In this study, we applied an ICEES functionality that allows users to run multiple chi-square comparisons based on available features and a primary outcome measure, with options to include a correction metric for multiple comparisons or collapse contiguous bins. In all cases, missing data were excluded from analysis. We queried the ICEES OpenAPI for data on all patients included in the asthma cohort and focused on outcomes in year 2016, which was the most recent year available with complete exposures data. We ran separate queries for each of the following primary outcome measures: (1) 1 or more annual ED or inpatient visits for respiratory issues; and (2) 1 or more annual prescriptions for prednisone. Specifically, we asked the following natural language question: “Among all patients within the ICEES asthma cohort, what airborne pollutant exposures, demographic features, and socioeconomic exposures differ significantly between patients with 0 versus 1 or more annual ED or inpatient visits for respiratory issues in year 2016?” The corresponding command-line API request was:

curl -X POST “https://icees.renci.org:16340/patient/2016/cohort/ COHORT%3A12/associations_to_all_features” -H “accept: text/tabular” -H “Content-Type: application/json” -d “{\”feature\“:{\”TotalEDInpatientVisits\“:{\”operator\“:\”=\“, \”value\“:0}},\”maximum_p_value\“:1}”

A similar query was used to examine the primary outcome of 1 or more annual prescriptions for prednisone.

### Statistical Analysis

The exploratory 1 × N feature association functionality available via the ICEES OpenAPI automatically invoked a chi-square test of the association between available features and our user-defined primary outcome measure, significance level, and multiple-comparison correction. We considered the primary outcomes of 1 or more annual ED or inpatient visits for respiratory issues and 1 or more annual prescriptions for prednisone. We focused our analysis on select feature variables that were considered a priori to have a potential impact on asthma exacerbations and were available for patients within the asthma cohort: demographic factors (age, sex, and race); socioeconomic exposures (residential density, health insurance access, and median household income); and airborne pollutant exposures (proximity to major roadway or highway, and exposure to PM_2.5_ and ozone). We set the significance level at α=.05, which was adjusted by Bonferroni correction to α=.001. A power calculation was not conducted, as this was an observational, exploratory study focused on existing biomedical data.

## Results

We successfully queried the ICEES OpenAPI for outcomes data on year 2016. Of the 157,410 patients who met the criteria used to define the asthma cohort, 26,332 patients (16.73%) had 1 or more annual ED or inpatient visits for respiratory issues, and 17,056 patients (10.84%) had 1 or more annual prescriptions for prednisone. Table 1 provides additional details on the cohort, including demographic and clinical profile and environmental exposures.

**Table 1:** Patient characteristics: demographic factors, environmental exposures, and clinical outcomes (N=157,410).

Feature variable		Values, n (%)
**Age at study start (years)**		
	<5	5638 (3.58)
	5-17	20,071 (12.75)
	18-44	35,777 (22.73)
	45-64	51,495 (32.71)
	65-89	44,429 (28.23)
**Sex**		
	Male	67,875 (43.12)
	Female	89,531 (56.88)
	Missing^a^/other	4 (<.0001)
**Race**		
	Caucasian	78,418 (49.82)
	African American	28,977 (18.41)
	Asian	1608 (1.02)
	American/Alaskan Native	902 (0.57)
	Native Hawaiian/Pacific Islander	67 (0.04)
	Unknown/other/missing	47,438 (30.14)
**Ethnicity**		
	Hispanic	7488 (4.76)
	Not Hispanic	105,925 (67.29)
	Unknown/missing	43,997 (27.95)
**Estimated residential density**		
	Rural area	95,632 (60.75)
	Urban cluster	41,798 (26.55)
	Urbanized area	0 (0)
	Missing	19,980 (12.69)
**Estimated probability of no health insurance**		
	[0, 0.0637]	34,500 (21.92)
	(0.0637, 0.1121]	34,313 (21.80)
	(0.1121, 0.1644]	34,340 (21.82)
	(0.1644, 0.5548]	34,201 (21.73)
	Missing	20,056 (12.74)
**Estimated median household income (US $)**		
	(7470, 36,635)	26,967 (17.13)
	(3663, 46,750]	27,081 (17.20)
	(46,750, 59,566]	26,843 (17.05)
	(59,566, 78,355]	26,968 (17.13)
	(78,355, 250,001]	26,955 (17.12)
	Missing	22,596 (14.35)
**Proximity to major roadway/highway (m)**		
	0-49	17,485 (11.11)
	50-99	9754 (6.20)
	100-149	10,244 (6.51)
	150-199	9398 (5.97)
	200-249	8477 (5.39)
	≥250	85,989 (54.63)
	Missing	46,694 (29.66)
**Average daily exposure to PM_2.5_ (μg/m^3^)^b^**		
	[3.27, 6.30]	8806 (5.59)
	(6.30, 7.81]	108,847 (69.15)
	(7.81. 10.83]	23,359 (14.84)
	Missing	16,398 (10.42)
**Maximum daily exposure to ozone (ppb)^b^**		
	[27.80, 39.00]	11,608 (7.37)
	(39.00, 42.73]	127,202 (80.81)
	(42.73, 46.45]	2202 (1.40)
	Missing	16,398 (10.42)
**Annual ED or inpatient visits for respiratory issues**		
	0	131,078 (83.27)
	≥1	26,332 (16.73)
**Annual prednisone prescriptions/administrations**		
	0	140,354 (89.16)
	≥1	17,056 (10.84)

^a^Missing data reflect gaps in the electronic health record data, particularly missing geocodes that prevented the determination of exposure estimates.

^b^Averaged over the 1-year study period.

**Figure 1:**
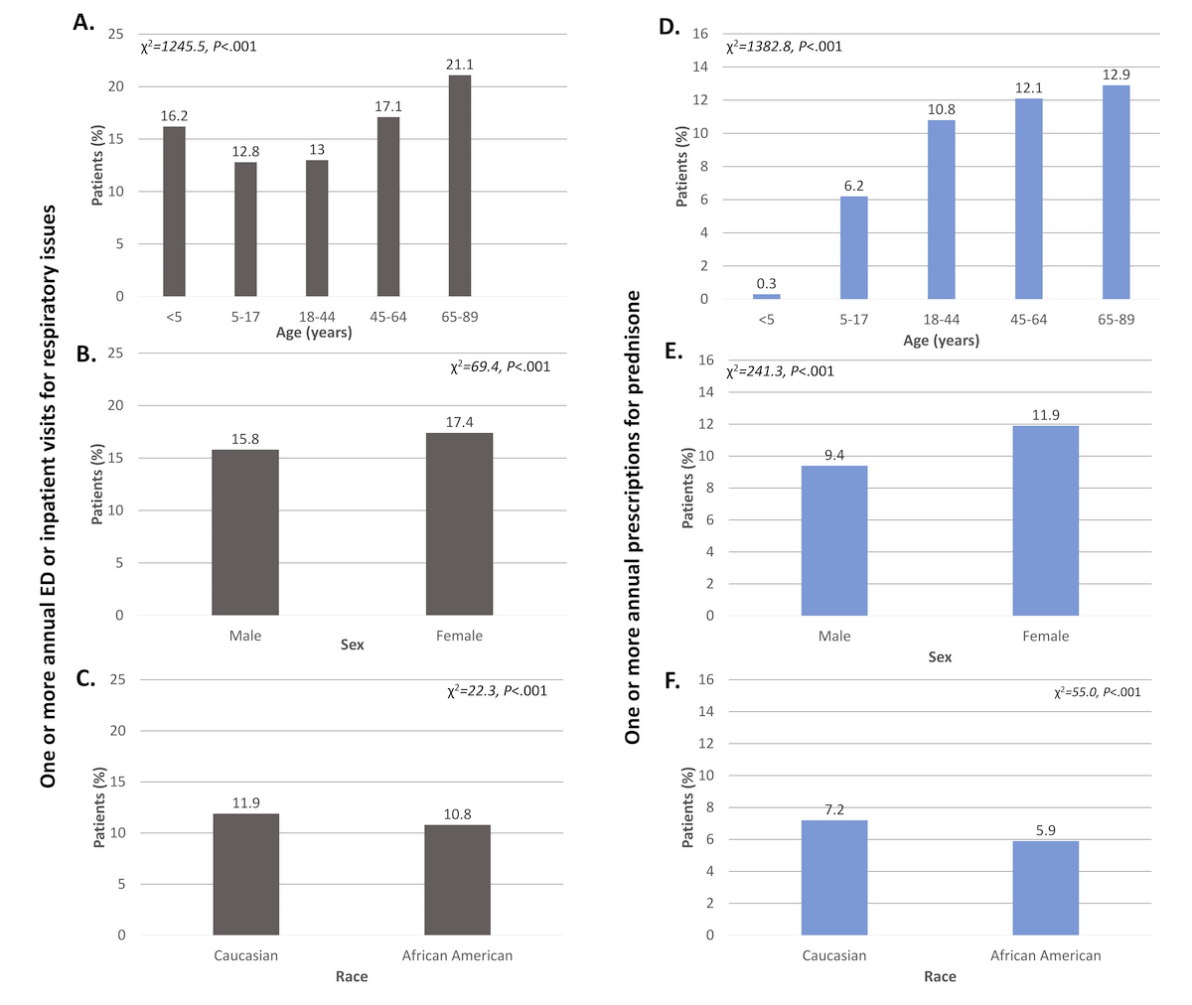
Associations between age (A,D), sex (B,E), race (C,F) and asthma exacerbations, defined as one or more annual emergency department (ED) or inpatient visits for respiratory issues (A-C) or one or more annual prescriptions for prednisone (D-F) (N = 157,410).

We then examined associations between select feature variables and annual ED or inpatient visits for respiratory issues, focusing initially on demographic factors (Figure 1A-C). We found that the percentage of patients with asthma exacerbations was higher among females than males (17.41% [15,587/89,531] vs 15.83% [10,743/67,875]; χ^2^=69.4; *P*<.001) and among Caucasians than African Americans (11.86% [9304/78,418] vs 10.83% [3137/28,977]; χ^2^=22.3; *P*<.001). A U-shaped relationship was found between age and the percentage of patients with 1 or more ED or inpatient visits for respiratory issues (<5 years, 16.21% [914/5638]; 5-17 years, 12.83% [2576/20,071]; 18-44 years, 13.01% [4655/35,777]; 45-64 years, 17.13% [8822/51,495]; 65-89 years, 21.08% [9365/44,429]; χ^2^=1245.5; *P*<.001). (Degrees of freedom are not returned by ICEES and thus are not reported here.)

**Figure 2:**
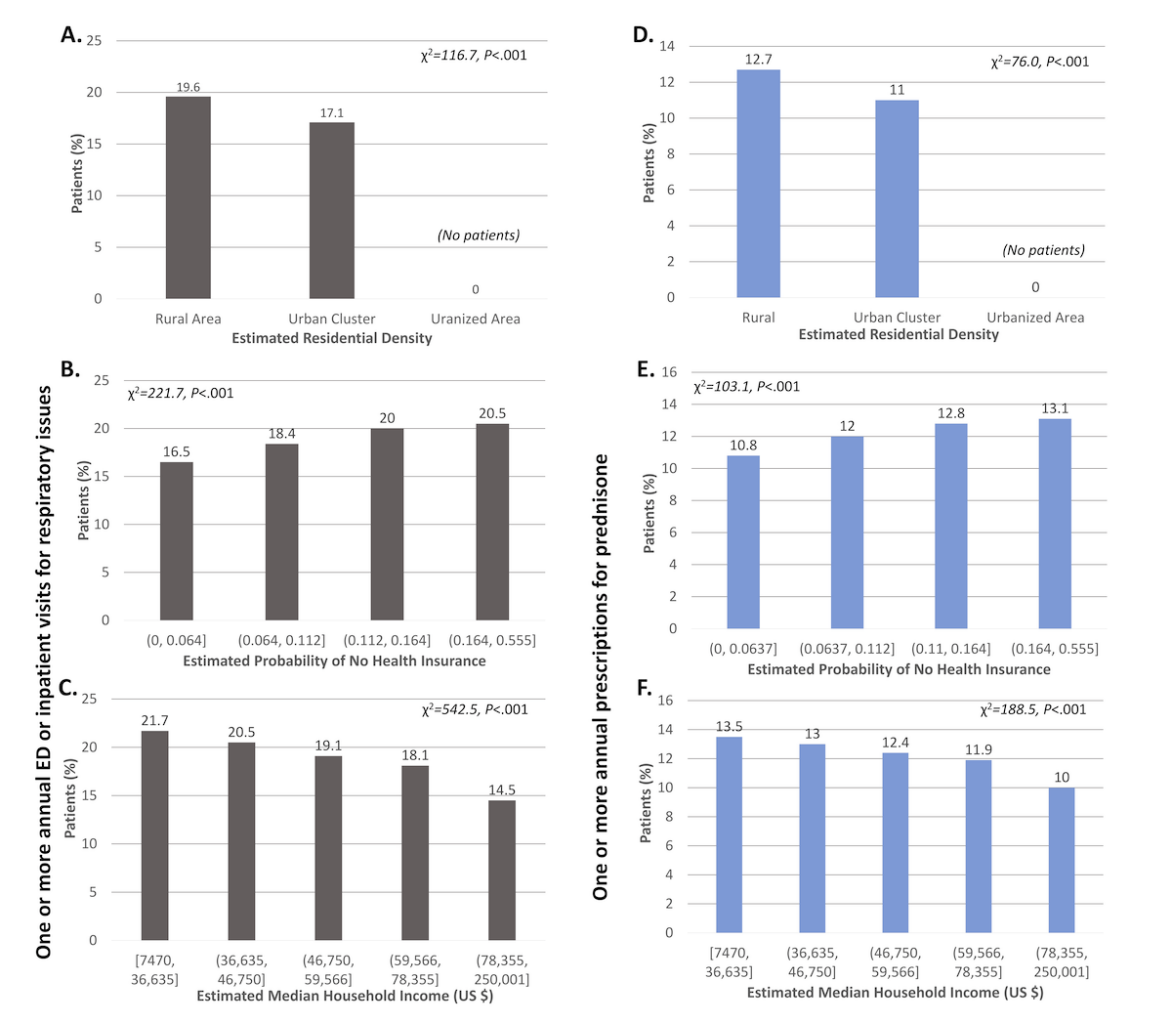
Associations between residential density (A,D), probability of no health insurance (B,E), and median household income (C,F) and asthma exacerbations, defined as one or more annual emergency department (ED) or inpatient visits for respiratory issues (A-C) or one or more annual prescriptions for prednisone (D-F) (N = 157,410).

We also examined associations between socioeconomic exposures and annual ED or inpatient visits for respiratory issues (Figure 2A-C). We found that the percentage of patients with 1 or more annual ED or inpatient visits for respiratory issues was higher among patients residing in low-density rural areas than among those residing in higher-density urban clusters (19.59% [18,739/95,632] vs 17.12% [7155/41,798]; χ^2^=116.7; *P*<.001). No patients were identified as residing in urbanized areas, as estimated by the American Community Survey and classified by the US Census Bureau. Asthma exacerbations increased with increasing probability of no health insurance (bin 1, 16.47% [5681/34,500]; bin 2, 18.43% [6325/34,313]; bin 3, 20.03% [6878/34,340]; bin 4, 20.47% [7000/34,201]; χ^2^=221.7; *P*<.001) and decreased with increasing median household income (bin 1, 21.68% [5847/26,967]; bin 2, 20.51% [5553/27,081]; bin 3, 19.10% [5127/26,843]; bin 4, 18.07% [4872/26,968]; bin 5, 14.46% [3898/26,955]; χ^2^=542.5; *P*<.001). (Bin values are provided in Table 1.)

**Figure 3:**
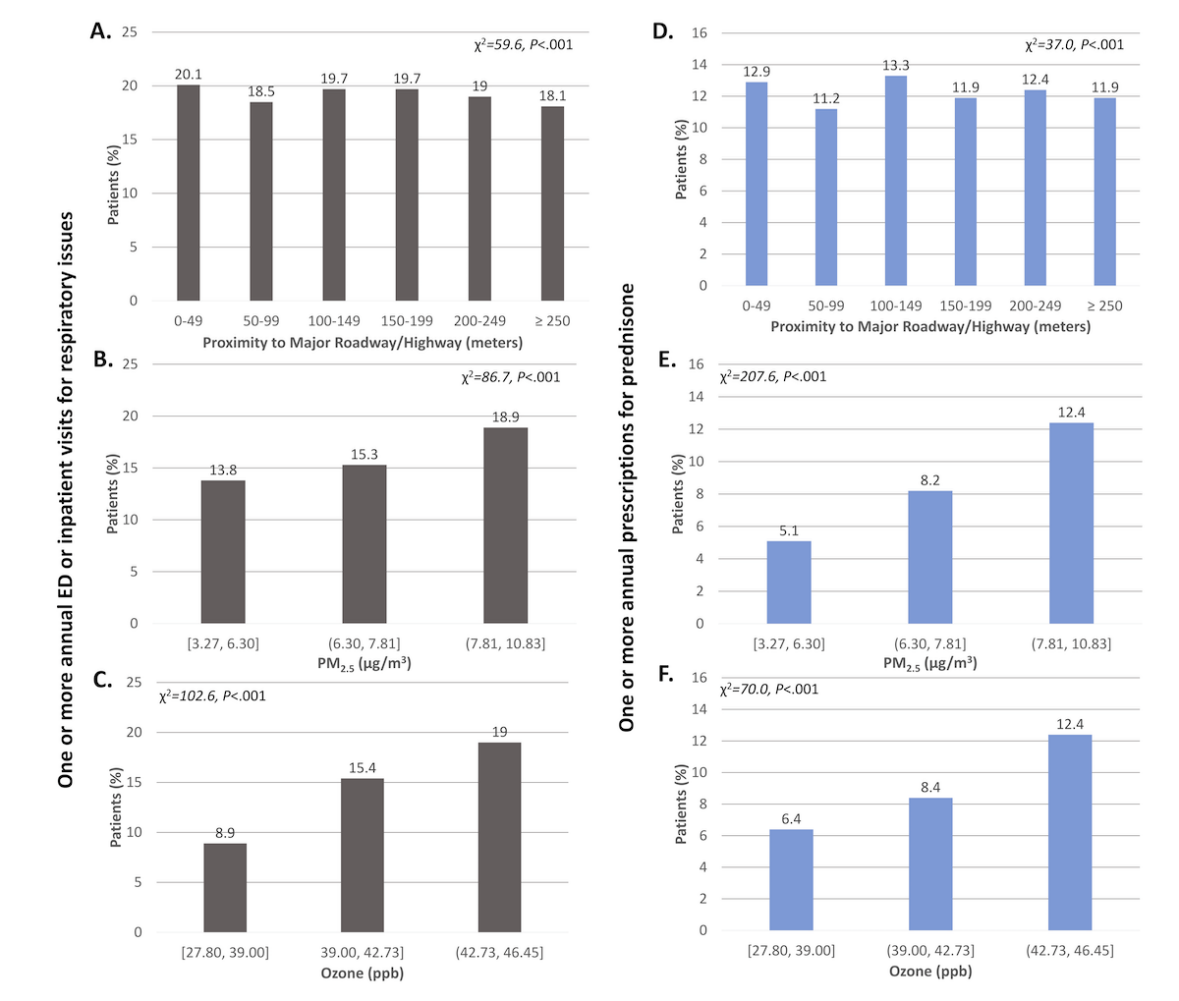
Associations between proximity to a major roadway or highway (A,D), exposure to particulate matter ≤ 2.5-μm (PM2.5) (B,E), exposure to ozone (C,F) and asthma exacerbations, defined as one or more annual emergency department (ED) or inpatient visits for respiratory issues (A-C) or one or more annual prescriptions for prednisone (D-F). (N = 157,410).

We then examined associations between airborne pollutant exposures and annual ED or inpatient visits for respiratory issues (Figure 3A-C). Asthma exacerbations decreased with increasing household distance from a major roadway or highway (0-49 m, 20.11% [3516/17,485]; 50-99 m, 18.45% [1800/9754]; 100-149 m, 19.73% [2021/10,244]; 150-199 m, 19.69% [1850/9398]; 200-249 m, 18.96% [1607/8477]; ≥250 m, 18.06% [15,529/85,989]; χ^2^=59.6; *P*<.001) and increased with exposure to increasing levels of PM_2.5_ (bin 1, 13.82% [300/2170]; bin 2, 15.33% [1017/6636]; bin 3, 18.89% [24,975/132,206]; χ^2^=86.7; *P*<.001) and ozone (bin 1, 8.90% [21/236]; bin 2, 15.38% [1749/11,372]; bin 3, 18.95% [24,522/129,404]; χ^2^=102.6; *P*<.001).

Results for the primary outcome of annual prescriptions for prednisone (Figures 1D-F, 2D-F, and 3D-F) were similar to those for annual ED or inpatient visits for respiratory issues, with 1 exception. Specifically, a linear relationship was found between age and prednisone prescriptions, with the percentage of patients with 1 or more annual prednisone prescription increasing with age (<5 years, 0.27% [15/5638]; 5-17 years, 6.1% [1235/20,071]; 18-44 years, 10.79% [3861/35,777]; 45-64 years, 12.08% [6222/51,495]; 65-89 years, 12.88% [5723/44,429]; χ^2^=1382.8; *P*<.001).

## Discussion

### Principal Findings

We describe the further development and application of ICEES+ to explore select feature variables associated with asthma exacerbations in a large cohort of patients with asthma or a related condition. We focused on select demographic factors, socioeconomic exposures, and airborne pollutant exposures. We compared results for 2 outcome measures that are indicative of asthma exacerbations: annual ED or inpatient visits for respiratory issues and annual prescriptions for prednisone. We found that female sex, Caucasian race, rural residential density, high probability of no health insurance, low estimated median household income, close residential proximity to a major roadway or highway, and exposure to relatively high levels of PM_2.5_ or ozone were significantly associated with asthma exacerbations. Moreover, the results were largely consistent across outcome measures, even though rates of annual ED/inpatient visits for respiratory issues were higher than those for annual prednisone prescriptions.

### Limitations

Our study has several limitations that should be considered when interpreting the results. Specifically, as an open service that exposes EHR data, ICEES must abide by stringent regulatory and institutional regulations that limit the granularity of data that can be exposed and the statistical capabilities that are supported. For instance, ICEES exposes binned or recoded data, not raw data. In addition, our institution treats exposure estimates as secondary PHI because they are derived from primary PHI (ie, geocodes and dates); as such, we are unable to reveal the estimated values themselves, only the bins, thus preventing a determination of mean exposures and other statistics based on continuous values. Finally, ICEES currently only supports basic bivariate statistical capabilities. However, we are developing approaches to adapt ICEES to support, in a regulatory-compliant manner, more sophisticated multivariate statistical approaches and machine learning algorithms [15,16].

### Comparison With Prior Work

We highlight several scientific findings and discuss unexpected findings. First, we observed an increase in the proportion of asthma exacerbations among females versus males. Asthma and acute exacerbations of asthma are more common in males than females in childhood. However, in adulthood, the effect of sex shifts, with females accounting for the majority of asthma and asthma exacerbations. As the majority of patients in our cohort were adults, this observation is consistent with what has been reported in the literature [17-19]. In addition, the increase in asthma exacerbations among patients with lower median household income and those lacking health insurance reflects established disparities in asthma management, particularly among minorities [20]. However, the increase in the proportion of asthma exacerbations among Caucasians versus African Americans was unexpected and contradicts both our findings [10] and those of other investigators [21]. While the reason for this apparent discrepancy is unclear, several possible explanations exist, including the fact that our institution’s racial category of “Caucasian” does not definitively distinguish Hispanic Caucasians from non-Hispanic Caucasians, which may have introduced variability. We are currently exploring approaches that may allow us to clearly distinguish Hispanic and non-Hispanic Caucasians and thus refine our racial and ethnic categorization. Another possible explanation is that our prior study focused on year 2010 [10], whereas this study focused on year 2016, and our institution’s demographics and patient catchment area have changed significantly over that period [22].

Second, the relationship between age and asthma exacerbations was U-shaped when based on annual ED or inpatient visits for respiratory issues and linear when based on annual prescriptions for prednisone. We suspect that this difference is due to the heterogeneity of wheezing phenotypes in the younger age range, which can be associated with different long-term prognoses for the development of asthma and variance in the use of oral corticosteroids for disease exacerbation [23-26].

Third, one of the key features of ICEES is that it supports research on the impact of environmental exposures such as airborne pollutants on health and disease. Indeed, we identified that asthma exacerbations increased with increasing exposure to PM_2.5_ and ozone, as we and others have shown [5,27]. We also found an increase in asthma exacerbations among patients residing in close proximity to a major roadway or highway, as others have found when using roadway exposure as a proxy for airborne pollutant exposure [14,28,29], although the effect in this study was modest. While one might have expected an increase in asthma exacerbations among persons living in densely populated areas, we found the opposite to be true, with increased asthma exacerbations among persons residing in low-density regions classified by the US Census Bureau as rural areas versus higher-density regions classified as urban clusters. We suspect that several factors might explain these findings. For instance, UNC Health’s patient catchment area draws heavily from rural regions of North Carolina, with multiple clinics and small hospitals located across the state and many patients relying on the state hospital system for health care services. Indeed, not a single patient in the cohort described in this study resided in a region classified by the US Census Bureau as an urbanized area. This may have introduced bias into the results. In addition, we note that many major roadways and highways run through rural parts of our patient catchment area, and so any presumption that close proximity to a major roadway or highway is more common in urban versus rural regions may not be valid. A related point is that rural exposures carry risks that may differ from urban exposures. For instance, we are expanding ICEES to include data on concentrated animal farming operations and landfills so that we can begin to examine exposures that may uniquely impact persons residing in rural regions.

We also highlight key technical aspects of this study and discuss limitations. First, the data reported herein are openly available via the ICEES OpenAPI, without any regulatory restrictions or login credentials. This allowed us to rapidly execute the queries and analyze the results, thereby accelerating the speed of discovery. Because ICEES is designed to be disease agnostic and is not restricted to patients with asthma and related conditions, we can adapt our approach and the service itself to support any number of use cases and explore environmental influences on virtually any disease. Indeed, we have deployed additional ICEES instances that expose data on patients with drug-induced liver injury and patients with coronavirus infection. In addition, we are adapting ICEES to support a use case on primary ciliary dyskinesia and related rare pulmonary disorders.

Second, by using health care system EHR data, a large and clinically relevant patient sample can be identified. In this study, our sample size was approximately 160,000 patients, thus supporting rigorous open statistical analysis. While the statistical tests available via the ICEES+ OpenAPI are currently limited to bivariate analyses, we are developing approaches to support multivariate analyses such as generalized linear models, random forest trees, and causal inference models, with options to control for potential covariates, account for missing data, and examine only those patients who are active in a given year, meaning that they were seen at 1 or more clinics within UNC Health. One significant challenge is the binning approach that is adopted for variables. For instance, automated binning algorithms typically bin data by value or by frequency. The former supports the study of extreme values, but at the expense of evenly distributed bin sizes; the latter supports an even distribution of observations among cells, but at the expense of overlap in patients with equal exposures between bins and bin cutoff points that may not be scientifically meaningful. We are systematically exploring this issue.

### Conclusions

Our results demonstrate that the open-source ICEES can be used to replicate and extend published findings on factors that influence asthma exacerbations. While we are actively researching the limitations of the service and developing ways to improve it, we believe that ICEES will greatly speed and democratize the use of EHR data to support research and discovery. Moreover, to the best of our knowledge, ICEES is the only open source of clinical data that have been integrated at the patient level with multiple sources of public environmental exposures data. While we have described an application use case focused on asthma, ICEES is disease agnostic. We expect the service to advance research in environmental health and related fields and continue to grow as we expand both our user base and the service itself to support new clinical use cases, additional EHR elements (eg, laboratory measures), and new data sources (eg, survey data). Moreover, because ICEES is open source, the model and software code [30,31] can be adopted by other institutions as a novel approach for openly exposing and sharing sensitive data. Indeed, ICEES may have application as an open, privacy-preserving approach to inform decision making by the US Environmental Protection Agency and other federal agencies regarding the patient-level impact of environmental exposures on risk of disease. Finally, we are assessing regulatory-compliant options for applying ICEES as a tool for clinical decision support by identifying patients with asthma (and eventually patients with other chronic diseases) or geographical regions at high risk for poor health outcomes based on their exposures profile and then flagging those patients in their EHR to inform patient care.

## Abbreviations

CAMP FHIR: Clinical Asset Mapping Program for Health Level 7 Fast Healthcare Interoperability Resource
ED: emergency department
EHR: electronic health record
FHIR PIT: Fast Healthcare Interoperability Resource Patient data Integration Tool
HIPAA: Health Insurance Portability and Accountability Act
ICEES: Integrated Clinical and Environmental Exposures Service
OPENAPI: open application programming interface
PHI: protected health information
PM: particulate matter ≤2.5 μm
